# Adaptive stochastic resonance for unknown and variable input signals

**DOI:** 10.1038/s41598-017-02644-w

**Published:** 2017-05-26

**Authors:** Patrick Krauss, Claus Metzner, Achim Schilling, Christian Schütz, Konstantin Tziridis, Ben Fabry, Holger Schulze

**Affiliations:** 1 Department of Otorhinolaryngology, University Erlangen, Nürnberg, Germany; 2 Department of Physics, University Erlangen, Nürnberg, Germany

## Abstract

All sensors have a threshold, defined by the smallest signal amplitude that can be detected. The detection of sub-threshold signals, however, is possible by using the principle of stochastic resonance, where noise is added to the input signal so that it randomly exceeds the sensor threshold. The choice of an optimal noise level that maximizes the mutual information between sensor input and output, however, requires knowledge of the input signal, which is not available in most practical applications. Here we demonstrate that the autocorrelation of the sensor output alone is sufficient to find this optimal noise level. Furthermore, we demonstrate numerically and analytically the equivalence of the traditional mutual information approach and our autocorrelation approach for a range of model systems. We furthermore show how the level of added noise can be continuously adapted even to highly variable, unknown input signals via a feedback loop. Finally, we present evidence that adaptive stochastic resonance based on the autocorrelation of the sensor output may be a fundamental principle in neuronal systems.

## Introduction

Biological organisms, as well as technical systems, rely on sensors that transmit environmental signals into the system for subsequent information processing. Sensors, in general, have a limited sensitivity, so that input signals with amplitudes below a certain threshold cannot normally be detected. Stochastic resonance (SR), a phenomenon first described by Benzi *et al*. in 1981^1^ enables non-linear systems to detect even sub-threshold signals by means of added noise^2–4^, which brings the weak input signal at random time points to above-threshold levels. This mechanism, however, requires a tuning of the added noise level to work properly. In the case of input signals with known properties, such as periodic signals with known frequency, the optimum noise level can be found by maximizing the signal-to-noise ratio of the sensor output^4, 5^. For arbitrary, non-periodic input signals, however, it is not possible to separate signal and noise in the detector output by frequency filtering, so that the quality of the signal transmission must be assessed in a different way.

Regardless of the detailed mechanism, SR has been identified in a wide range of biological systems, including the mating behavior of *Nezara viridula* that are able to detect subthreshold acoustic calling signals when mixed with acoustic Gaussian noise of suitable intensitiy^6^, rat cutaneous mechanoreceptors when stimulated with subthreshold aperiodic stimuli plus noise^2^ and the paddlefish which relies on electrical signals, amplified with stochastic resonance, to hunt edible plankton^7^. SR has recently received increasing attention especially in the context of experimental and computational neuroscience where it helps to explain how neuronal systems operate in noisy environments^8–10^.

**Figure 1:**
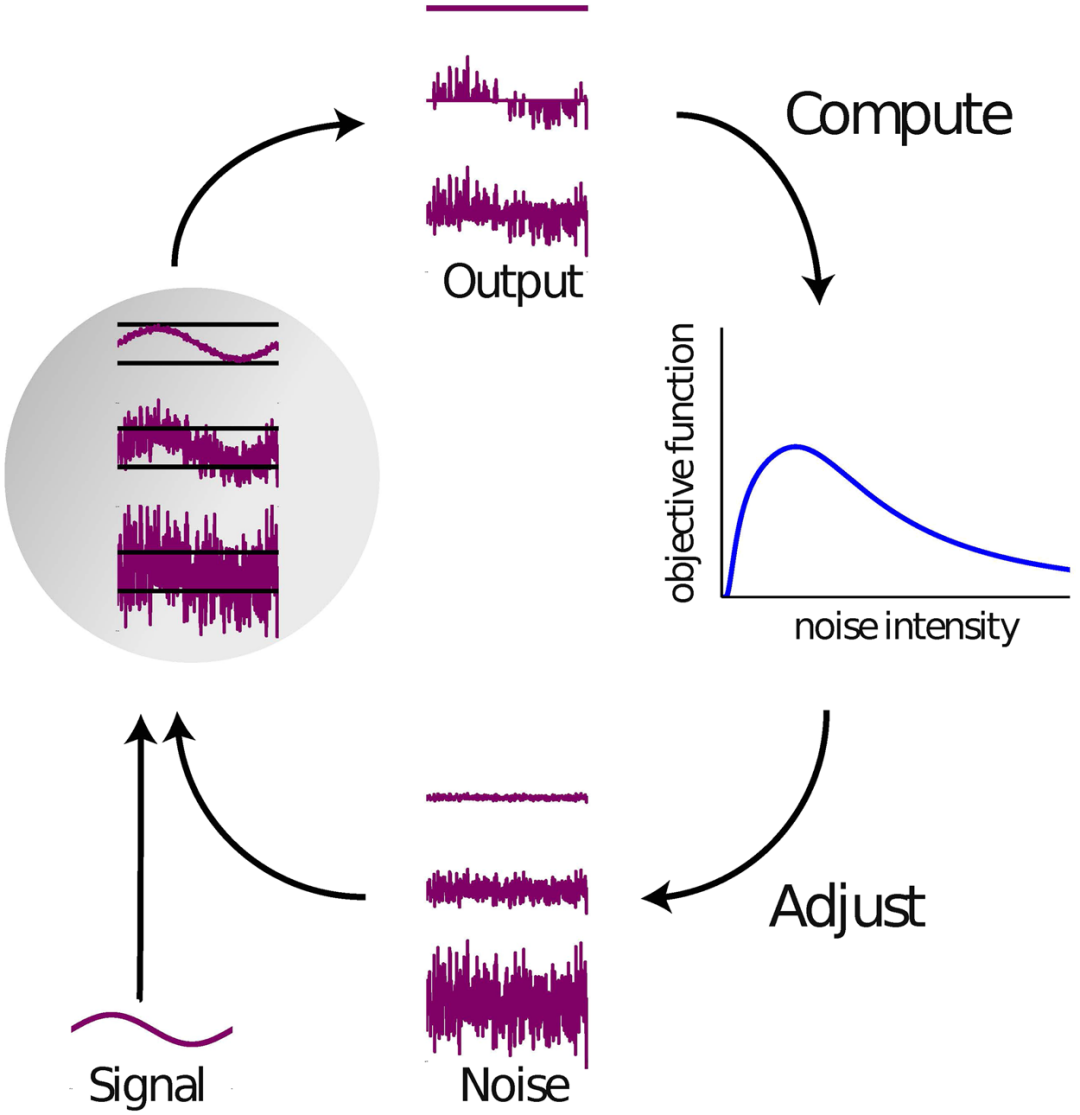
Principle of adaptive SR. Stochastic resonance describes how a sub-threshold signal with added noise of sufficient amplitude can result in a non-zero sensor output. If the input signal amplitude changes over time, the optimal noise level can be estimated and adjusted in a feedback loop from a resonance curve. If the probability density of the input signal levels is known, the resonance curve can be obtained from a variety of objective functions. For unknown input signals, however, the only applicable objective function is the autocorrelation of the sensor output.

It can be demonstrated that an optimal level for the added noise exists that maximizes the information transmission from the sub-threshold input to the sensor output^11, 12^. In self-adaptive non-linear signal detection systems based on SR, the optimum noise level can be continuously adjusted (increased or reduced until an optimum is found) via a feed-back loop, so that the system response in terms of information throughput remains optimal even if the properties of the input signal change (Fig. 1). For this processing principle, the term adaptive SR has been coined^13–15^.

A widely used measure (in the following refered to as ‘objective function’) for quantifying the information throughput is the mutual information (MI, cf. Methods) between the input signal and the sensor output^3, 12, 14^ a statistical quantity that measures the mutual dependence of these two variables^16^. It has been shown previously that the MI has a maximum at a well-defined, optimal intensity of the added noise^12^.

To calculate the MI (or other objective functions such as the signal-to-noise ratio, or the cross-correlation between input and output signal), the input signal must be known^2–4, 6, 10–13, 17^, but this is often not the case. Here, we consider the practically important case of unknown and not necessarily periodic input signals. Although finding the optimal level of noise becomes less important with arrays of transducers^17^, it still remains an unsolved problem for single detector systems.

In this letter we show that this fundamental limitation of adaptive SR can be overcome by another objective function, namely the autocorrelation (AC, cf. Methods) of the detector output. Maximizing the output AC leads to similar or identical estimates of optimal noise intensities for SR as estimates based on established objective functions, yet with the decisive advantage that no knowledge of the input signal is required (Fig. 1).

## Results

**Figure 2:**
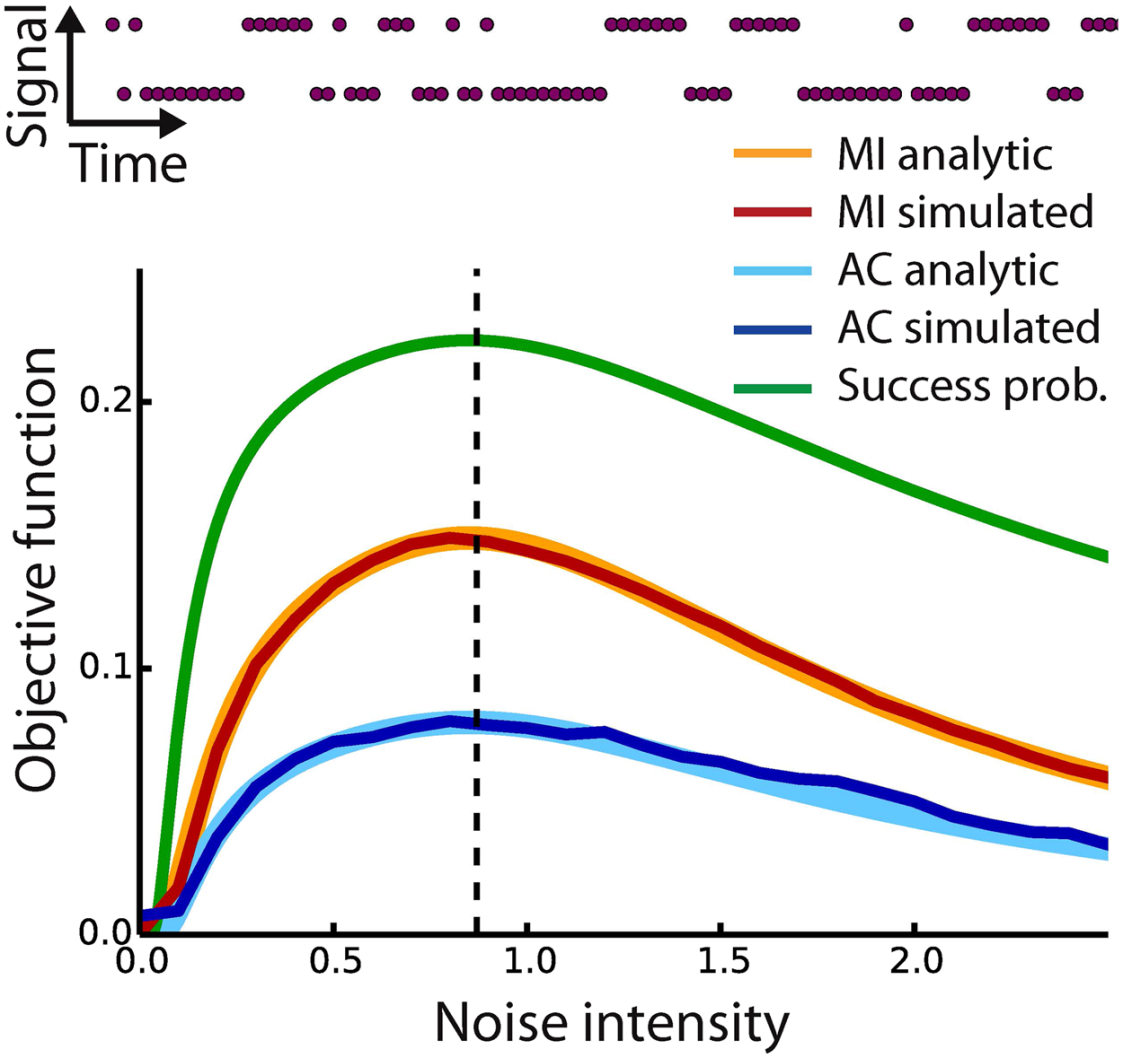
Results of analytical model and numerical simulations. Top: Example of a discrete signal consisting of a correlated bipolar string with *p*(*s*_*t*_ = *s*_*t*+1_) = 0.7. Bottom: Comparison of different objective functions versus noise intensity for correlated bipolar strings with symmetric threshold and a signal-to-threshold distance of 0.1. Analytical and numerical solutions coincide, and all objectives functions show a maximum at the same noise level. MI = mutual information, AC = autocorrelation of the output signal.

To demonstrate the equivalence of the mutual information (cf. Methods) between input and output, and the autocorrelation of output (cf. Methods), for finding the optimal noise level, we consider a so-called discrete-symmetric model with a symmetric threshold |*θ*| = 1.1 and a discrete output *y*_*t*_ = {−1.0, 0.0, 1.0}, i.e. the sensor output is zero for a sensor input *s*_*t*_ of −*θ* < *s*_*t*_ < *θ*, −1.0 for *s*_*t*_ < −*θ*, and 1.0 for *s*_*t*_ > *θ*. As a discrete input signal we generate a correlated bipolar string *s*_*t*_ = {−1.0, 1.0} with the probability of successive values being identical *p*(*s*_*t*_ = *s*_*t*+1_) = 0.7 (Fig. 2 top). Without added noise, the input signal alone never passes the thresholds.

Furthermore, we introduce the concept of the success probability (cf. Supplements): i.e. the conditional probability that, given a certain input, the correct corresponding output is produced. For a discrete-symmetric sensor with correlated bipolar input, we prove analytically (cf. Supplements) that the success probability as a function of noise intensity has a well-defined peak, indicating the existence of an optimal level of noise for SR. We also prove that the mutual information and the autocorrelation of the output can be expressed as strictly monotonous functions of the success probability (cf. Supplements). Hence both, the mutual information and the autocorrelation of the output exhibit their maximum at the same level of noise. We numerically tested our approach and confirmed that both the mutual information and the autocorrelation of the output yielded similar optimal noise levels (Fig. 2 bottom). In addition, we applied our analytical model to the important case of soft thresholds^18^ and non-Gaussian noise and show that this only changes the success probability *Q*, whereas the MI and AC remain monotonous functions of *Q* and, hence, peak at the same level of noise (cf. Supplements).

To generalize our finding and demonstrate that both approaches for quantifying the information throughput give similar optimal noise intensities, we simulated a variety of models comprising different discrete and continuous input signals, different types of detector models and different signal-to-threshold distances (the difference between the temporal averaged sub-threshold signal and the threshold).

**Figure 3:**
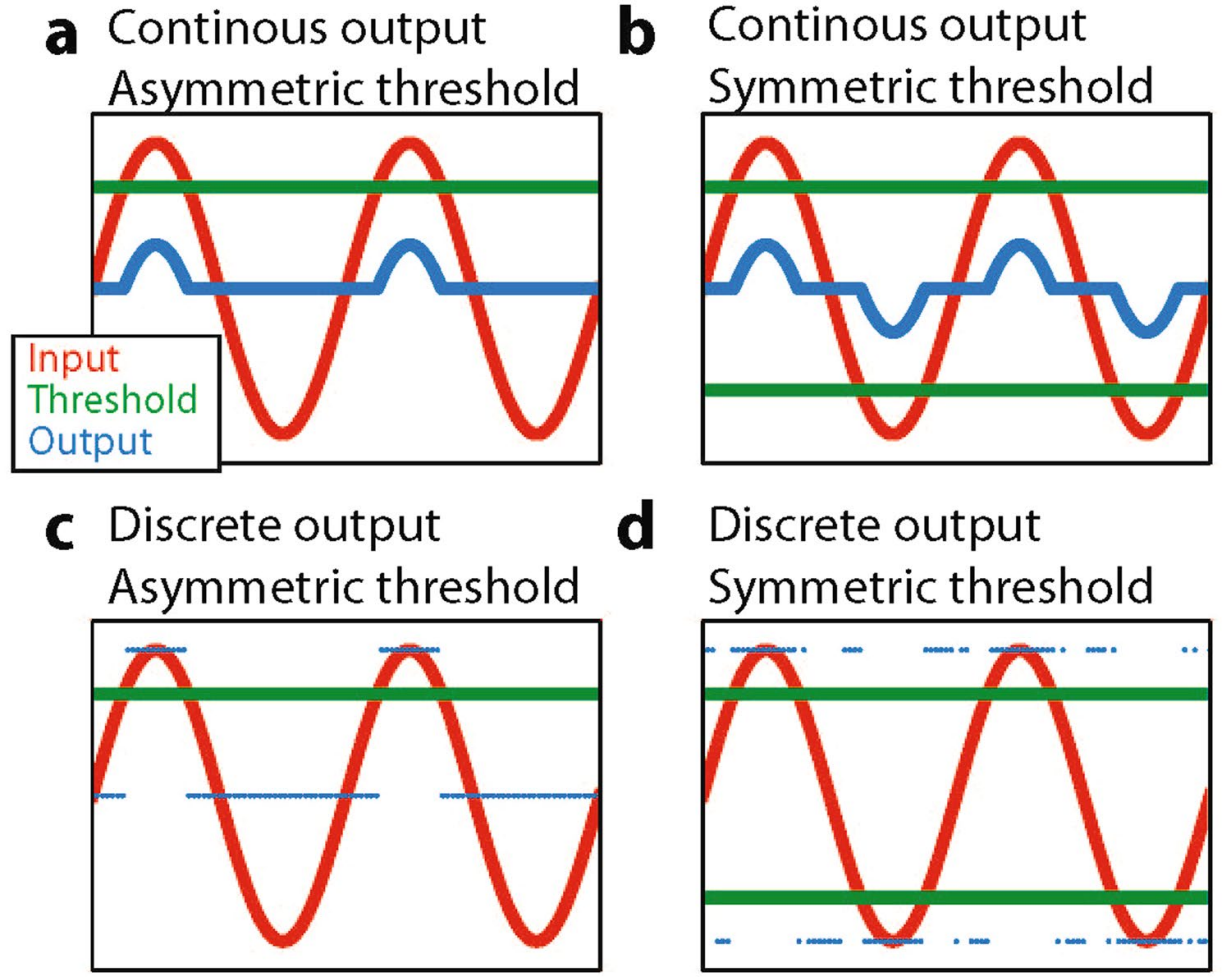
Detector models. Sketch, demonstrating the reponse of different sensor types to a sinusoidal input siganl (red line). We compare the four cases of continuous (**a**,**b**) or discrete (**c**,**d**) output (blue lines or dots, respectively) and with asymmetric (**a**,**c**) or symmetric (**b**,**d**) thresholds (green lines).

As detectors, we tested the above described discrete-symmetric model (Fig. 3d), a discrete-asymmetric (Fig. 3c), a continuous-asymmetric (Fig. 3a) and a continuous-symmetric (Fig. 3b) detector model (cf. Methods). These detectors are all memoryless, i.e. their output at a certain time depends only on their input at that time but neither on their internal state, nor on past inputs. We also tested a model with memory and implemented the biologically important neuronal leaky integrate-and-fire model (cf. Methods).

**Figure 4:**
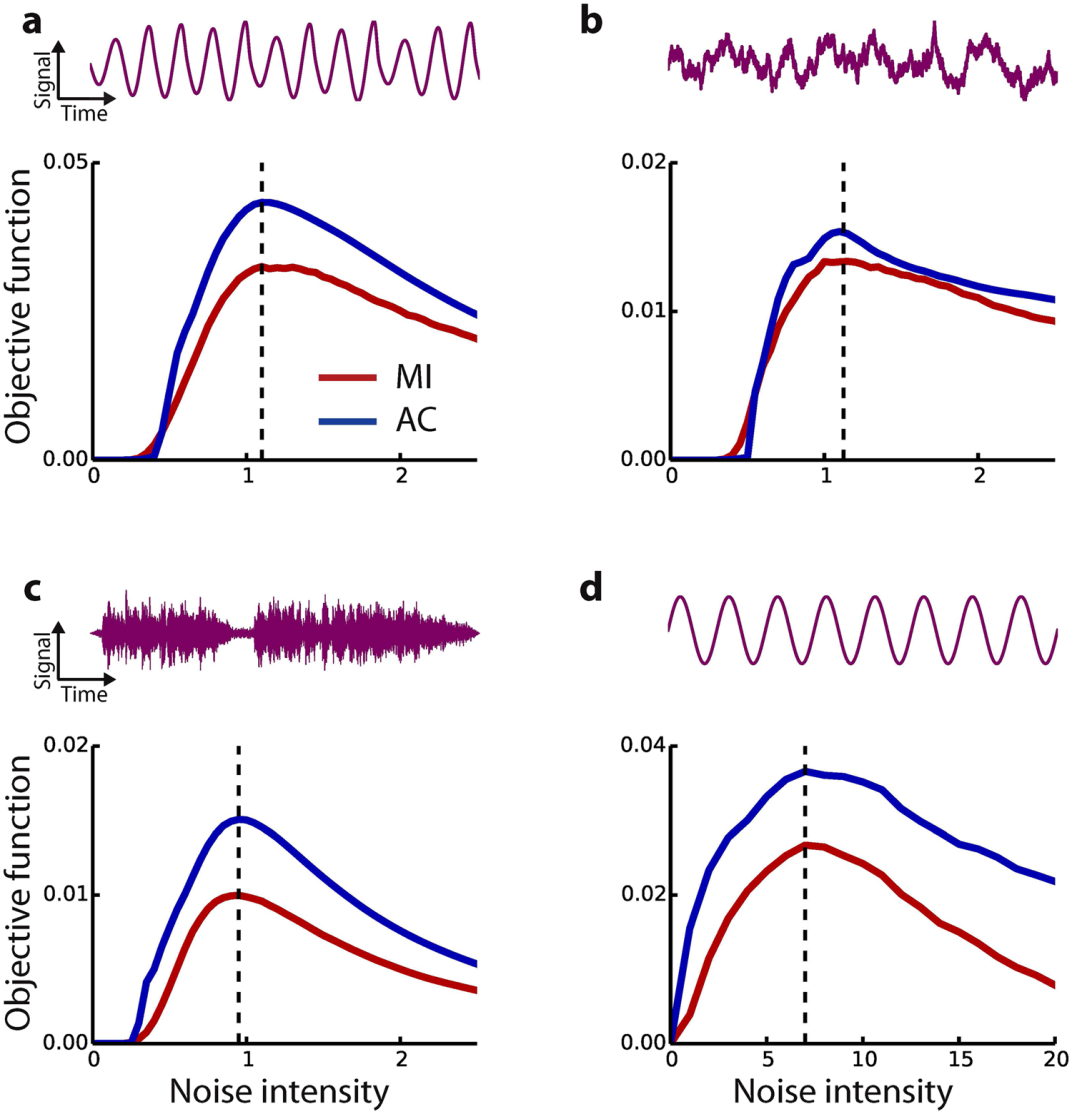
Performance of the output autocorrelation method. Comparison of resonance curves using the mutual information (red) and the output autocorrelation (blue) for different continuous signals. (**a**) Projection of the 3-dimensional Roessler attractor in one dimension, asymmetric threshold. (**b**) Ornstein-Uhlenbeck process, symmetric threshold. (**c**) Wave files of recorded speech, music and natural sounds, symmetric threshold. The biologically important case of the leaky integrate-and-fire model is shown in (**d**) with sine wave with constant frequency and amplitude as input. The signal-to-threshold distance was 0.1 for all cases.

As discrete aperiodic input signals, we used the correlated bipolar string described above (Fig. 2 top). In addition, we tested different time series of continuous aperiodic signals, namely the time series x(t) of the 3-dimensional Roessler attractor (Fig. 4a top, cf. Methods), an Ornstein-Uhlenbeck process (Fig. 4b top, cf. Methods), and wave files of recorded speech (Fig 4c top). Furthermore, a sine wave with constant frequency and amplitude was used as continuous periodic input (Fig. 4d top).

Four sample resonance curves (i.e. the mutual information and the autocorrelation of the output as functions of noise intensity) are shown in Fig. 4. In all cases the mean signal-to-threshold distance in arbitrary units was set to 1.1. Although the resonance curves from the mutual information (red) and the autocorrelation of the output (blue) are different, they peak at identical noise intensities.

**Figure 5:**
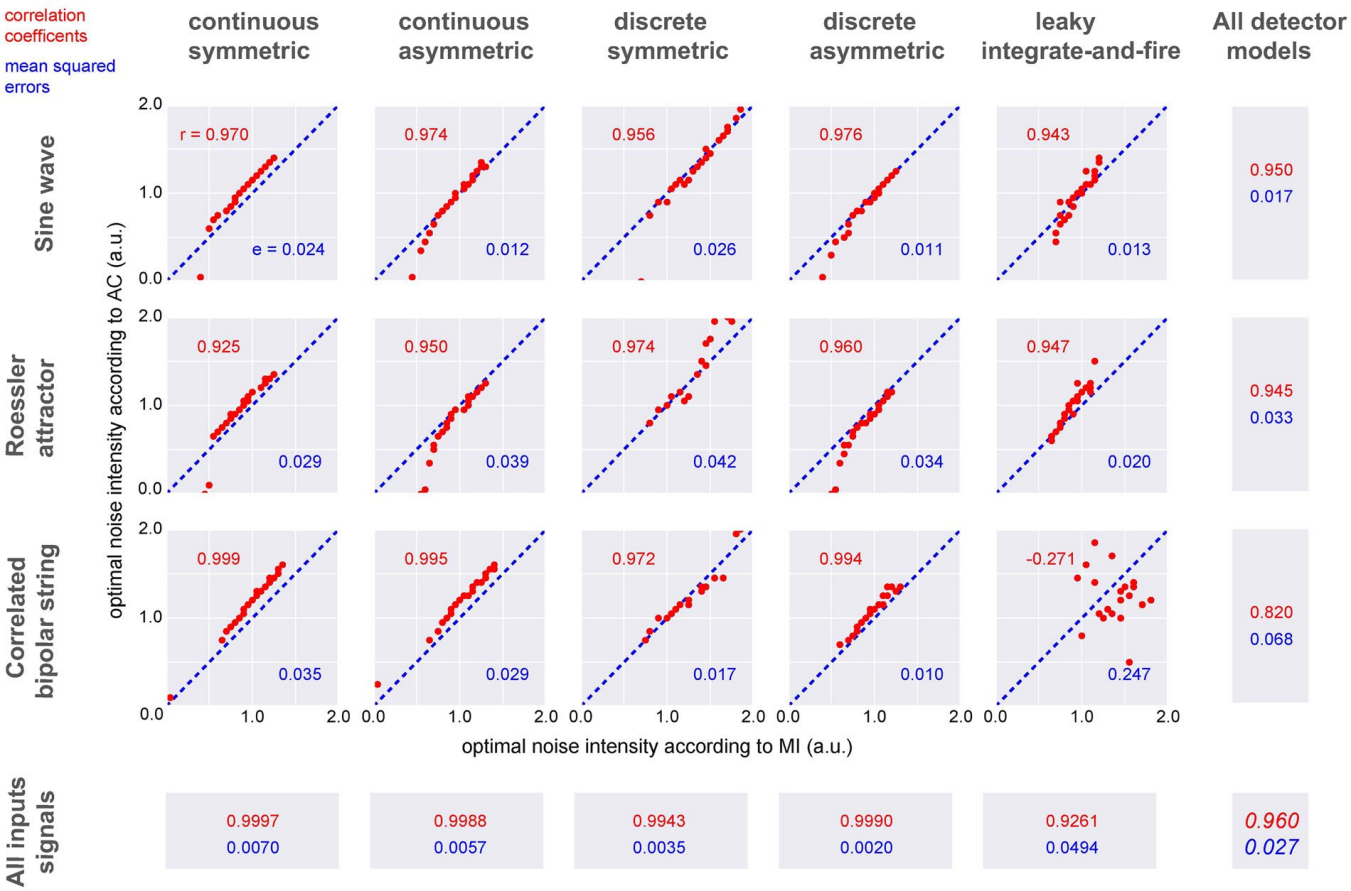
Correlation between optimal noise level from mutual information versus output autocorrlation. Numerical data from 5 sensor models, 3 different input signal, and different signal-to-threshold distances. The optimal noise intensities found by maximizing the mutual information (x-axes) are plotted versus the optimal noise intensities found by maximizing the output autocorrelation (y-axes). Except for correlated bipolar strings in combination with a leaky integrate-and-fire sensor, the optimal noise levels from both methods are highly correlated, with an overall correlation coefficient of *r* = 0.960 and a mean squared error of *e* = 0.027.

We then tested all combinations of the five different detector models (that is: continuous-symmetric, continuous-asymmetric, discrete-symmetric, discrete-asymmetric, leaky integrate-and-fire) and the three input signal types (correlated bipolar string, Roessler attractor, sine wave) with twenty different signal-to-threshold distances (from 0.0 to 1.0 in steps of 0.05, arbitrary units) (Fig. 5). For each combination, ten trials were performed with different seeds for the random number generator resulting in a total of 3000 simulations. For each simulation, the optimal noise intensities according to the mutual information (x-axes) and the autocorrelation of the output (y-axes) have been evaluated and averaged over the ten performed trials per combination. Almost all tested combinations yielded nearly identical estimates of optimal noise intensity. The total correlation coefficient between optimal noise intensity based on mutual information versus autocorrelation was *r* = 0.96 with a mean squared error of *e* = 0.026. Except for the combination of a correlated bipolar string with the leaky integrate-and-fire neuron, all other combinations yielded correlation coefficients from 0.925 to 0.999 with mean squared errors from 0.010 to 0.042, further demonstrating the equivalence of the two measures.

## Discussion

We have shown that the autocorrelation of the output signal can serve as a universal objective function to estimate the optimal level of noise in SR-based sensory systems. This new approach allows for the first time the technical implementation of adaptive SR in situations where the information content of the input signal is unknown or highly variable. Once the signal is optimally transmitted by the sensor, the (noisy) output signal can be further improved by several techniques, such as kernel density estimation^19^.

Several interesting phenomena are closely related to stochastic resonance. A well-known technical system to reduce the effect of noise during analog-to-digital conversion is the Schmitt trigger^20^, whereby two different analog thresholds are defined for the rising and falling edges of the discretized signal. For the case of a periodic input signal and colored Gaussian noise, the ocurrance of SR in this system has been demonstrated analytically^21^.

In dithering^22^, noise is added to audio or image signals in order to reduce the perception of quantization errors and the appearance of spurious large-scale patterns that can result from analog-to-digital conversion. Such spurious large scale patterns are associated with positive autocorrelations in the digitized signal. Therefore, although the aim in dithering is not to increase information transmission, the subjective perception of the signal by a human recipient may be improved by adding noise that in this case reduces such positive autocorrelations.

Related to this is the phenomenon of ghost stochastic resonance, which emerges when nonlinear systems are driven by periodic signals with more than one frequency in the presence of noise. An example for this effect is the auditory perception of ‘ghost’ frequencies that are not present in the acoustic pressure wave. The uncovering of this phenomenon helped to understand the perception of pitch in complex sound signals and the so-called ‘missing fundamental illusion’^23, 24^. Interestingly, when noise of increasing intensity is added to sound, the emergence of a ghost resonance coincides with a maximum of the output autocorrelation^23, 24^. This, in turn is closely linked to the effect of coherence resonance^25^, whereby a non-linear excitable system is driven by noise, resulting in a maximally autocorrelated system output at a certain optimum noise intensity.

Although SR can explain a number of adaptive processes in neural systems, in particular in auditory and visual signal processing^26^, how exactly that may be implemented is a matter of debate. Even for known input signals, the use of the mutual information or related approaches seem daunting for a biological system, since calculating them requires mathematical operations that are hard to implement in neuronal networks. By contrast, the autocorrelation function can be easily implemented with neuronal networks using delay-lines and coincidence detectors, as proposed in ref. 27 and verified, for example, in the nucleus laminaris of birds (barn owls), where they serve to code interaural time differences^28^. More recent work has shown that a surrogate of the autocorrelation function can even be computed by a single noisy neuron^23, 24^. Therefore, adaptive SR based on output autocorrelation may be a major processing principle in neural sensory systems and perception, and may also be responsible for pathologic conditions including neuropathic pain or tinnitus^26^.

## Methods

### Mutual Information

The mutual information *I*(*S*; *Y*) quantifies the mutual dependence of two random variables *S* and *Y*.^16^. It determines how similar the joint distribution *p*(*s*, *y*) is compared to the product of the marginal distributions *p*(*s*)*p*(*y*)

1 $$I(S;Y)=\sum _{s}\sum _{y}\,p(s,y)\,{\mathrm{log}}_{2}(\frac{p(s,y)}{p(s)p(y)}),$$

For continuous random variables, the summation is replaced by a double integral:

2 $$I(S;Y)={\int }_{S}{\int }_{Y}p(s,y){\mathrm{log}}_{2}(\frac{p(s,y)}{p(s)\,p(y)})\,dy\,ds,$$

where *p*(*s*, *y*) is now the joint probability density function of *S* and *Y*, and *p*(*s*) and *p*(*y*) are the marginal probability density functions. The natural unit of *I*(*S*; *Y*) is *bits*, however in some cases it is more convenient to divide the total mutual information by the time, or by the number of spikes within the observed spike train, and thus derive mutual information rates *R*(*S*; *Y*) measured in *bits s* ^−1^ or *bits spike* ^−1^. The choice of the mutual information as an objective function for adaptive stochastic resonance is natural, because the fundamental purpose of any sensor is to transmit information into a subsequent information processing system. Indeed, it has been shown by several authors^3, 12, 14^ that, within the context of stochastic resonance, *I*(*S*; *Y*) as a function of the variance *σ* ^2^ of the added noise has a maximum that indicates the optimal level of noise.

### Output autocorrelation

The output autocorrelation function of the time lag *τ* (more precisely, the autocorrelation coefficient^29^) is defined as

3 $${C}_{yy}(\tau )=\frac{{\langle ({y}_{t+\tau }-\bar{y})({y}_{t}-\bar{y})\rangle }_{t}}{{\langle {({y}_{t}-\bar{y})}^{2}\rangle }_{t}},$$

where $\overline{y}$ is the mean and 〈⋅〉_*t*_ indicates averaging over time. We note that for most applications (and discrete time steps) it is sufficient to consider only one time lag, i.e. *τ* = 1. However, for more complex signals, e.g. streams of n-bit words, it might be beneficial to calculate *C*_*yy*_(*τ*) for a number of different subsequent lag times. In order to derive a single objective value in the case of multiple time lags *τ*, the root mean square (rms^29^) of the autocorrelation function

4 $$\,{\rm{R}}{\rm{M}}{\rm{S}}\,({C}_{yy})=\sqrt{\frac{1}{{N}_{\tau }}\sum _{\tau }{({C}_{yy}(\tau ))}^{2}}$$

is calculated, where *N*_*τ*_ is the total number of different time lages.

### Input signals for numerical simulations

Both, synthetically generated as well as natural signals were used in numerical simulations. As an example of a discrete input signal, a correlated, bipolar string *s*_*t*_ ∈ {−1, +1} was generated, in which the probability of successive values being identical was Prob(*s*_*t*_ = *s*_*t*−1_)  = 0.7. As examples of continuous signals we used: first, a sine waveform signal with constant frequency and amplitude; second, an aperiodic time series derived from the variable *x*(*t*) of the Roessler attractor^30^

5 $$\begin{array}{rcl}\dot{x} & = & -(y+z)\\ \dot{y} & = & x+ay\\ \dot{z} & = & b+(x-c)z,\end{array}$$

with parameters *a* = 0.15, *b* = 0.2 and *c* = 7.1; third, the aperiodic random Ornstein-Uhlenbeck process^31^ $\dot{x}=-\,\frac{1}{\tau }x+\epsilon \,\xi (t)$, where *ξ* is an independent normally distributed random variable, *τ* is the correlation time and $\epsilon$ is the noise amplitude; fourth, wave files of speech, music and natural sounds. All synthetically generated signals were computed by numerically integrating the differential equations using fourth order Runge Kutta method.

### Sensor models for numerical simulations

Four different memory-less sensor models were implemented by combining symmetric and asymmetric thresholds, with discrete and continuous sensor output functions (Fig. 5). In the symmetric models, there exist two thresholds +*θ* and −*θ*. Without added noise, the sensor output is zero for |*s*_*t*_| < *θ*, *s*_*t*_ − *θ* for *s*_*t*_ > *θ*, and *s*_*t*_ + *θ* for *s*_*t*_ < −*θ*. In the asymmetric models, there exists only a single positive threshold *θ* > 0. Here, without added noise, the sensor output is zero for *s*_*t*_ < *θ* and *s*_*t*_ − *θ* for *s*_*t*_ >= *θ*. We note that the analytical model described above belongs to the class of discrete symmetric models. As one example of the detectors with memory, were the output depends not only on the momentary input and added noise, but also on earlier internal states of the detector itself, we choose the leaky integrate-and-fire neuron model^32, 33^ with

$$\dot{x}=-\,\frac{1}{{\tau }_{m}}x+{s}_{t}$$

where *x* is the membrane potential, *τ*_*m*_ the mebrane time constant and *s*_*t*_ the input signal. If *x* crosses the threshold *θ* from below, an output spike is generated and the membrane potential is set to the resting potential *x*_*r*_, which is chosen to be zero for simplicity.

## Electronic supplementary material

Supplements
