# Supplementary material for: Adaptive stochastic resonance for unknown and variable input signals
Source: Sci Rep. 2017 May 26;7:2450. doi: 10.1038/s41598-017-02644-w (PMC5446399; doi:10.1038/s41598-017-02644-w)
Supplement: Supplementary file 1 — Supplements [file 41598_2017_2644_MOESM1_ESM.pdf]

Adaptive stochastic resonance for unknown and variable input signals

Patrick Krauss[1,2], Claus Metzner[2], Achim Schilling[1,2], Christian Schütz[1], Konstantin Tziridis[1], Ben Fabry[2] and Holger Schulze[1,3]

[1]Experimental Otolaryngology, ENT-Hospital, Head and Neck Surgery, Friedrich-Alexander University Erlangen-Nürnberg (FAU), Germany
[2]Department of Physics, Center for Medical Physics and Technology, Biophysics Group, Friedrich-Alexander University Erlangen-Nürnberg (FAU), Germany
[3]Correspondence to holger.schulze@uk-erlangen.de

April 5, 2017

Supplements

Success probability The output of a memory-less sensor can be described by a conditional probability distribution $p(y_t|s_t, n_t)$, which includes deterministic behaviour as a special case. Assuming statistically independent noise with distribution $p(n_t)$, the signal transmission properties of the sensor are given by $p(y_t|s_t) = \sum_{n_t} p(y_t|s_t, n_t)p(n_t)$. Ideally, the sensor output should be equal to the input signal, $y_t = s_t$, so that $p(y_t|s_t) = \delta_{y_t,s_t}$. It is therefore meaningful to quantify the performance of a sensor by the success probability

$$Q = p(y_t = s_t), \tag{1}$$

which is expected to peak at some optimum noise level within the context of stochastic resonance.

Analytical model In general, the momentary response of a SR-sensor can depend on the history of internal states of the system, as is the case in integrate-and-fire-neurons. For simplicity, in the analytical model we only consider memory-less sensors, which respond to the present input signal s_t and noise value n_t independently from their former activity states.

We consider a bipolar stochastic sensor in which both the input signal s_t and the sensor output y_t can only take on the values -1 and $+1$. The noise values n_t, however, are continuous gaussian random numbers with variance σ^2 and mean $\mu = 0$, without any temporal correlations. We further assume that these two values appear in the input signal with equal probalility, $p(s_t = -1) = p(s_t = +1) = 0.5$. By assuming two symmetric detection thresholds $\pm\theta$ (figure 3 lower right inset), together with symmetric white noise, it can be assured that the distribution of sensor outputs $p(y_t = -1) = p(y_t = +1) = 0.5$ is also symmetric, so that the mean, variance and entropy of y_t remain constant even if the noise level is changed. Hence, the expressions for $I(S;Y)$ and $C_{yy}(\tau)$ can be slightly simplified. In particular, the autocorrelation can be reduced to the non-normalized form $C_{yy}(\tau) \propto \langle y_t y_{t+\tau} \rangle$, and, furthermore, will be considered only for lagtime $\tau = 1$.

The sensor adds the noise n_t to the binary input signal s_t. If $s_t + n_t$ exceeds the upper threshold θ, the output y_t is $+1$, if $s_t + n_t$ falls below the lower threshold $-\theta$, output y_t is -1. For $s_t + n_t \in [-\theta, +\theta]$, the output is chosen randomly between the two binary values $+1$ and -1.

We are interested in the case of a threshold $\theta > 1$ which exceeds the signal amplitude, so that without the assistance of added noise the signal cannot be detected. Adding a random noise value n_t to a (say) positive input signal s_t can have three possible effects. If we consider the noise to be sufficiently positive to lift the signal beyond the upper threshold, then the success probability $Q = p(y_t = +1 \mid s_t = +1) = p(y_t = -1 \mid s_t = -1)$ will be increased. Alternatively, if the noise happens to be strongly negative and draws the positive signal below the lower threshold $-\theta$ then the success probability Q will be decreased. The third possibility is that $s_t + n_t$ remains sub-threshold. Such cases make the signal transmission neither better nor worse.

It is intuitively clear that small noise levels will increase Q, but as soon as a considerable fraction of momentary noise levels n_t exceeds $2+(\theta-1)$, the success probability Q will fall again. In our case it is given by $Q = Q(\sigma) = \frac{1}{2} + \frac{1}{2}\left[\, W(\frac{\theta+1}{\sigma}) - W(\frac{\theta-1}{\sigma})\,\right]$, where $W(x) = \frac{1}{2}\mathrm{erf}(\frac{x}{\sqrt{2}})$ is a slightly rescaled error function (see *Derivation of success probability* for a detailed derivation). As a function of the noise level σ, the success probability has a well-defined maximum.

In this sensor model, the mutual information $I(Y;S)$ can be expressed as a strictly increasing function of the success probability: $I(Q) = 1 + Q\log_2 Q + (1-Q)\log_2(1-Q)$ (see *Derivation of mutual information* for a detailed derivation).

Since both I and Q require access to the sub-threshold signal s_t, we turn to the autocorrelation function C_{yy} of the sensor output. Since the mean $\overline{y}$ of y_t is zero and its variance constant, we can use a non-normalized version of equation(6). Furthermore, we restrict our analytical consideration to a single lag-time $\tau = 1$, defining C $= \langle y_t y_{t+1}\rangle$. The modulus of this quantity, too, can be expressed as a strictly increasing function of the success probability: $|C(Q)| = |\,\langle s_t s_{t+1}\rangle\,|\;\;[1-4Q(1-Q)]$, where $\langle s_t s_{t+1}\rangle$ are the input correlations (see *Derivation of output autocorrelation* for a detailed derivation).

Derivation of success probability The normalized Gaussian distribution with zero-mean and standard deviation σ is given by

$$g(x,\sigma) = \frac{1}{\sqrt{2\pi}\sigma}\; e^{-\frac{1}{2}(x/\sigma)^2} \tag{2}$$

For later convenience, we define a function $W(x)$ via

$$W\left(\frac{z}{\sigma}\right) = \int_0^z g(x,\sigma)\;dx = \frac{1}{2}\;\mathrm{erf}\left(\frac{1}{\sqrt{2}}\;\frac{z}{\sigma}\right), \tag{3}$$

where $\mathrm{erf}(x) = \frac{2}{\sqrt{\pi}}\int_0^x e^{-t^2}dt$ is the error function.

The success probability Q is given by

$$\begin{aligned} Q &= p(y_t{=}{+1}|s_t{=}{+1}) = \\ &= \frac{1}{2}\cdot p(\;-\theta-1 < n_t < \theta-1\;) + \\ &+ \;\; p(\;n_t > \theta-1\;) \end{aligned} \tag{4}$$

The factor $\frac{1}{2}$ accounts for the stochastic output of the unit in the case when $s_t + n_t$ is sub-threshold. We can now express the probabilities as integrals over Gaussians:

$$
\begin{aligned}
Q &= \frac{1}{2}\cdot\left(\int_0^{\theta-1} g(x,\sigma)dx + \int_0^{\theta+1} g(x,\sigma)dx\right) + \\
&+ \left(\frac{1}{2} - \int_0^{\theta-1} g(x,\sigma)dx\right)
\end{aligned}
\tag{5}
$$

Next we use the function $W(x)$ defined above:

$$
\begin{aligned}
Q &= \frac{1}{2}\cdot\left(W\left(\frac{\theta-1}{\sigma}\right) + W\left(\frac{\theta+1}{\sigma}\right)\right) + \\
&+ \left(\frac{1}{2} - W\left(\frac{\theta-1}{\sigma}\right)\right) = \\
&= \frac{1}{2} + \left[\,W\left(\frac{\theta+1}{\sigma}\right) - W\left(\frac{\theta-1}{\sigma}\right)\,\right]
\end{aligned}
\tag{6}
$$

Derivation of mutual information The mutual information of the detector output and the input signal is defined as

$$
\begin{aligned}
I(Y;S) &= \sum_{y,s} p(y,s)\ \log_2\left(\frac{p(y,s)}{p(y)p(s)}\right) = \\
&= \sum_{y,s} p(y|s)p(s)\ \log_2\left(\frac{p(y|s)p(s)}{p(y)p(s)}\right) = \\
&= \sum_{y,s} p(y|s)(1/2)\ \log_2\left(\frac{p(y|s)(1/2)}{(1/2)(1/2)}\right) = \\
&= \frac{1}{2}\sum_{y,s} p(y|s)\ \log_2\left(2p(y|s)\right).
\end{aligned}
\tag{7}
$$

We explicitly go through all four terms:

$$
\begin{aligned}
2I(Y;S) &= \sum_{y,s} p(y|s)\ \log_2\left(2p(y|s)\right) = \\
&= p(y{=}-1|s{=}-1)\ \log_2\left(2p(y{=}-1|s{=}-1)\right) + \\
&+ p(y{=}-1|s{=}+1)\ \log_2\left(2p(y{=}-1|s{=}+1)\right) + \\
&+ p(y{=}+1|s{=}-1)\ \log_2\left(2p(y{=}+1|s{=}-1)\right) + \\
&+ p(y{=}+1|s{=}+1)\ \log_2\left(2p(y{=}+1|s{=}+1)\right) = \\
&= Q\ \log_2\left(2Q\right) + \\
&+ (1-Q)\ \log_2\left(2(1-Q)\right) + \\
&+ (1-Q)\ \log_2\left(2(1-Q)\right) + \\
&+ Q\ \log_2\left(2Q\right).
\end{aligned}
\tag{8}
$$

Therefore

$$
\begin{aligned}
I(Y;S) &= Q\ \log_2(2Q) + (1-Q)\ \log_2(2(1-Q)) \\
&= 1 + Q\ \log_2(Q) + (1-Q)\ \log_2(1-Q). \qquad (9)
\end{aligned}
$$

Derivation of output autocorrelations in the analytical model The temporal correlations of the input signal can be expressed by the probability $q = p(s_1 = +1, s_0 = +1)$ in the following way:

$$
\begin{aligned}
& \langle s_{t+1}\ s_t \rangle = \langle s_1\ s_0 \rangle = \\
= & \sum_{s_0, s_1} p(s_1, s_0)\ (s_1\ s_0) = \\
= & \ p(s_1 = -1 | s_0 = -1) p(s_0 = -1)\ [(-1)(-1)] + \\
+ & \ p(s_1 = -1 | s_0 = +1) p(s_0 = +1)\ [(-1)(+1)] + \\
+ & \ p(s_1 = +1 | s_0 = -1) p(s_0 = -1)\ [(+1)(-1)] + \\
+ & \ p(s_1 = +1 | s_0 = +1) p(s_0 = +1)\ [(+1)(+1)] = \\
= & \ q\ (1/2)\ [1] + \\
+ & \ (1-q)\ (1/2)\ [-1] + \\
+ & \ (1-q)\ (1/2)\ [-1] + \\
+ & \ q\ (1/2)\ [1] = 2q - 1. \qquad (10)
\end{aligned}
$$

The temporal correlations in the output signal are given by

$$
\begin{aligned}
C_{yy}(\tau = 1) &= \langle y_{t+1}\ y_t \rangle = \langle y_1\ y_0 \rangle = \\
&= \sum_{y_0, y_1} p(y_1, y_0)\ (y_1\ y_0). \qquad (11)
\end{aligned}
$$

Consider for example the probability $p(y_1 = +1, y_0 = +1)$. There are four different chains of events which can produce a sequence of two successive +1's in the output signal:

$$\begin{aligned}
& p(y_1 = +1, y_0 = +1) = \\
= \; & p(y_1 = +1 | s_1 = -1) p(s_1 = -1 | s_0 = -1) \cdot \\
\cdot \; & p(y_0 = +1 | s_0 = -1) p(s_0 = -1) + \\
+ \; & p(y_1 = +1 | s_1 = -1) p(s_1 = -1 | s_0 = +1) \cdot \\
\cdot \; & p(y_0 = +1 | s_0 = +1) p(s_0 = +1) + \\
+ \; & p(y_1 = +1 | s_1 = +1) p(s_1 = +1 | s_0 = -1) \cdot \\
\cdot \; & p(y_0 = +1 | s_0 = -1) p(s_0 = -1) + \\
+ \; & p(y_1 = +1 | s_1 = +1) p(s_1 = +1 | s_0 = +1) \cdot \\
\cdot \; & p(y_0 = +1 | s_0 = +1) p(s_0 = +1) = \\
= \; & (1 - Q) \; q \; (1 - Q) \; (1/2) + \\
+ \; & (1 - Q) \; (1 - q) \; Q \; (1/2) + \\
+ \; & Q \; (1 - q) \; (1 - Q) \; (1/2) + \\
+ \; & Q \; q \; Q \; (1/2) = \\
= \; & \frac{q}{2} + (2q - 1) Q (1 - Q) =: A. \qquad (12)
\end{aligned}$$

For symmetry reasons, $p(y_1 = -1, y_0 = -1) = p(y_1 = +1, y_0 = +1) = A$. In the same way, $p(y_1 = +1, y_0 = -1) = p(y_1 = -1, y_0 = +1) = B$.

Since $\sum_{y_0, y_1} p(y_1, y_0) = 1 = 2A + 2B$, it follows that $B = \frac{1}{2} - A$.

Knowing all four joint probabilities, we can proceed to compute the temporal correlations in the output signal:

$$\begin{aligned}
C_{yy}(\tau = 1) &= \langle y_1 \; y_0 \rangle = \\
&= A(-1)(-1) + B(-1)(+1) + \\
&+ B(+1)(-1) + A(+1)(+1) = \\
&= 2A - 2B = \\
&= (2q - 1) \, [\, 1 - 4Q(1 - Q) \,] = \\
&= \langle s_{t+1} \; s_t \rangle \; [\, 1 - 4Q(1 - Q) \,] \, . \qquad (13)
\end{aligned}$$

Soft thresholds and non-Gaussian noise As described above, the probabilistic information transmission from the signal input s_t to the output y_t of a sensor are defined by

$$p(y_t | s_t) = \int_{-\infty}^{+\infty} p(y_t | s_t, n_t) \; p(n_t) \; dn_t. \qquad (14)$$

Here, $p(y_t | s_t, n_t)$ characterizes the properties of a specific sensor type, and $p(n_t) = p_{noi}(n_t)$ is the *noise distribution*.

In this work, we are considering sensors where signal and noise are combined additively, so that $p(y_t|s_t, n_t)$ can be replaced by a simpler conditional probability that depends only on the sum $x_t = s_t + n_t$:

$$p(y_t|s_t, n_t) \longrightarrow p(\ y_t \mid s_t{+}n_t = x_t\ ) = p(y_t|x_t). \tag{15}$$

Furthermore, in the case of bipolar sensors, where $y_t = +1$ and $y_t = -1$ are the only possible outputs, the sensor can be characterized by a *response function*:

$$P_{res}(x) = p(y_t = +1|x_t = x). \tag{16}$$

For such additive, bipolar sensors, the success probability Q can be expressed via the response function and the noise distribution as

$$\begin{aligned} Q &= p(y_t = +1|s_t = +1) \\ &= \int_{-\infty}^{+\infty} P_{res}(x)\ p(n_t = x{-}1)\ dx \\ &= \int_{-\infty}^{+\infty} P_{res}(x)\ p_{noi}(x{-}1)\ dx. \end{aligned} \tag{17}$$

So far in this Supplemental, we have only considered detectors with a piecewise constant respose function (that is, where $P_{res}(x < -\theta) = 0$ and $P_{res}(-\theta <= x <= +\theta) = \frac{1}{2}$ and $P_{res}(x > +\theta) = 1$), and where the noise was normal distributed (see Fig.1(a)).

However, we can easily generalize our analytical model to permit arbitrary response functions (for example, by using a smooth sigmoidal function rather than one with hard thresholds) and non-Gaussian noise distributions. In order to keep the symmetry $p(y_t = -1) = p(y_t = +1) = 0.5$ of the output signals (which we have used to simplify our analytical derivation), we have to restrict our choices to response functions with $P_{res}(-x) = 1 - P_{res}(+x)$ and to noise distributions with $p_{noi}(-x) = p_{noi}(+x)$. One of the possible choices is sketched in Fig.1(b).

The generalization of the model to smooth sigmoidal sensor responses and non-Gaussian noise does only affect the success probability $Q = Q(\sigma)$ and its dependence on the noise amplitude σ. As long as $Q(\sigma)$ has a peak at some optimum noise level σ_{opt}, the strictly monotonous dependence of the mutual information I and the output correlations C_{yy} on Q guaranty that I and C_{yy} will peak at the same noise level σ_{opt}.

Different types of detectors and multiplicative noise For all bipolar sensors (with $s_t \in \{-1, +1\}$ and $y_t \in \{-1, +1\}$) a success probability can always be defined as

$$Q = \int_{-\infty}^{+\infty} p(y_t = +1 \mid s_t = +1,\ n_t)\ p(n_t)\ dn_t. \tag{18}$$

The general conditional probability $p(y_t = +1 \mid s_t = +1,\ n_t)$ includes not only detectors where signal and noise are combined additively, but allows for an arbitrary probabilistic dependence of y_t on s_t and n_t. As long as the noise n_t is temporally un-correlated (and all variables s_t, n_t and y_t are zero-mean) C_{yy} and the MI will remain monotonous functions of Q.

However, it is not guaranteed that these objective functions will always have a maximum as a function of the noise strength σ. Consider, for example, a sensor system where the zero-mean, temporally correlated signal s_t is *multiplied* with zero-mean, white noise n_t, and where the product $x_t = s_t * n_t$ is compared with a hard or soft sigmoidal threshold, as above. In this case, the amplitude modulated noise x_t is also un-correlated, so that $C_{yy} = 0$, no matter how the noise strength σ is set. Thus, for such multiplicative systems, the output auto-correlation is not in general a suitable objective function for adaptive SR. In the context of neural systems, however, the assumptions of a symmetric bipolar threshold and of completely un-correlated noise are not biologically plausible.

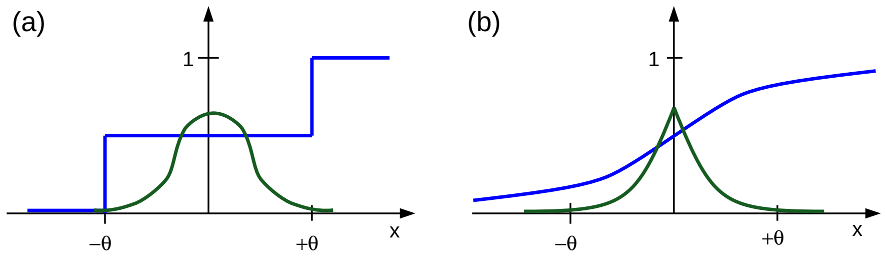

Figure 1: Sketch of possible response functions (blue) and noise distributions (green). (a) Piecewise constant $P_{res}(x)$ and Gaussian $p_{noi}(x)$. (b) Smooth sigmoidal $P_{res}(x)$ and leptocurtic $p_{noi}(x)$
